# L_1_*k-t* ESPIRiT: Accelerating Dynamic MRI Using Efficient Auto-Calibrated Parallel Imaging and Compressed Sensing Reconstruction

**DOI:** 10.1186/1532-429X-18-S1-P302

**Published:** 2016-01-27

**Authors:** Claudio Santelli, Sebastian Kozerke

**Affiliations:** grid.5801.c0000000121562780Institute for Biomedical Engineering, University and ETH Zurich, Zurich, Switzerland

## Background

Iterative self-consistent parallel imaging (PI) reconstruction (SPIRiT) [1, *Lustig M, MRM 64:457-71,2010*] has been extended for dynamic imaging by exploiting temporal correlations in *k-t* space (*k-t* SPIRiT) [2*,Santelli C, MRM 72:1233-45, 2014*]. Using eigendecomposition of a modified SPIRiT operator, computationally optimized reconstruction formally translates into auto-calibrated SENSE (ESPIRiT) [3, *Uecker, MRM 71:990-1001, 2014*]. In this work, this principle is applied to a *k-t* SPIRiT operator resulting in SENSE-like reconstruction of a coil-combined *x-f* space object. The method is tested on dynamic cardiac short-axis view data and compared to standard L_1_-regularized *k-t* SPIRiT.

## Methods

**Figure 1:**
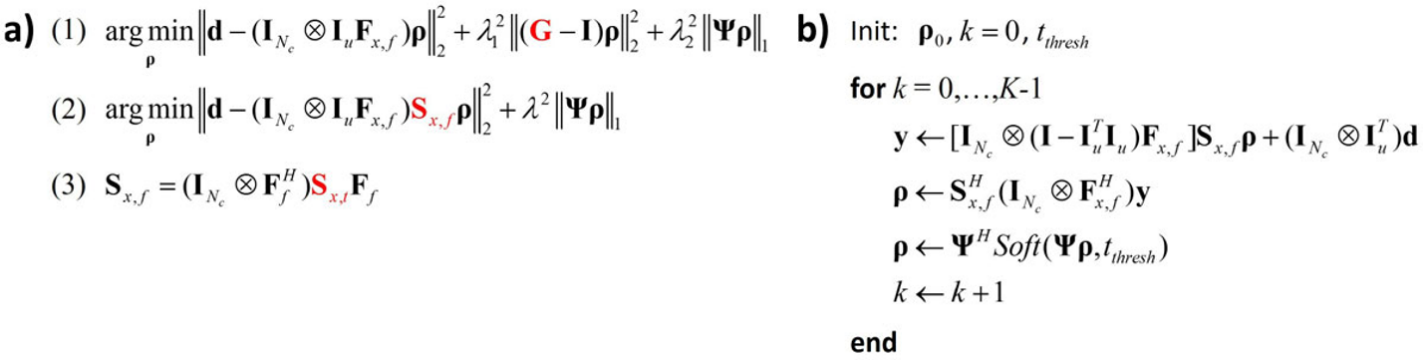
**a)** ***k-t*** **SPIRiT (1) and** ***k-t*** **ESPIRiT (2) minimization problems with PI operators G and S**_***x,f***_. While (1) solves for a multi-channel *x-f* object, (2) reconstructs a coil-combined image (**d**: *k-t* space data, **F**_*x,f*_: Fourier transform from *x-f* to *k-t* space, **I**_*u*_: undersampling matrix, λ's: regularization parameters). (3) shows the relation between **S**_*x,f*_and the temporally resolved coil sensitivities **S**_*x,t*_. **b)** L_1_ *k-t* ESPIRiT POCS reconstruction algorithm. Soft denotes the element-wise soft-thresholding operation.

L_1_*k-t* SPIRiT reconstructs a multi-coil *x-f* image series **ρ** by solving the optimization problem (1) (Figure 1a). For each *x-f* voxel, the PI-operator **G** reduces to a matrix-vector multiplication resulting into a computational complexity of *O*(*N*_*c*_x*N*_*c*_) (*N*_*c*_: No. of coils). Following [3], eigenvectors and eigenvalues of **G** assemble the matrix **S**_*x,f*_(composed of stacked diagonal matrices) directly transforming an *x-f* object into its multi-coil sensitivity-weighted representation. Thereby, the PI matrix **G** in (1) can be replaced by **S**_*x,f*_in the modified data-consistency term in (2) (Figure 1a). Solving (2), termed as L_1_*k-t* ESPIRiT, then results in a computationally optimized equivalent of (1) with an *O*(*N*_*c*_) PI operator and a sparsifying transform **Ψ** acting on a coil-combined image **ρ**. Similar to [4, *Lai P, ISMRM:345, 2010*], an algorithm to solve (2) for Cartesian random variable-density undersampling is given in Figure 1b.

Breath-held fully sampled cine 2D balanced SSFP short axis view data were acquired from a healthy subject on a 3T scanner (Philips Ingenia, Philips Healthcare, Best, The Netherlands). 28-channel data was compressed to 12 virtual channels [5, *Buehrer M, MRM(57):1131-39, 2007*]. **G** and **S**_*x,f*_were derived from the central k-space profiles of the 5-fold retrospectively decimated data. Due to the sparse *x-f* support, **Ψ** was set to identity **I**. *k-t* SPIRiT and *k-t* ESPIRiT reconstructions were both performed using POCS-like algorithms as described in [1] and Figure 1b, respectively (*K* = 30 iterations each).

## Results

**Figure 2:**
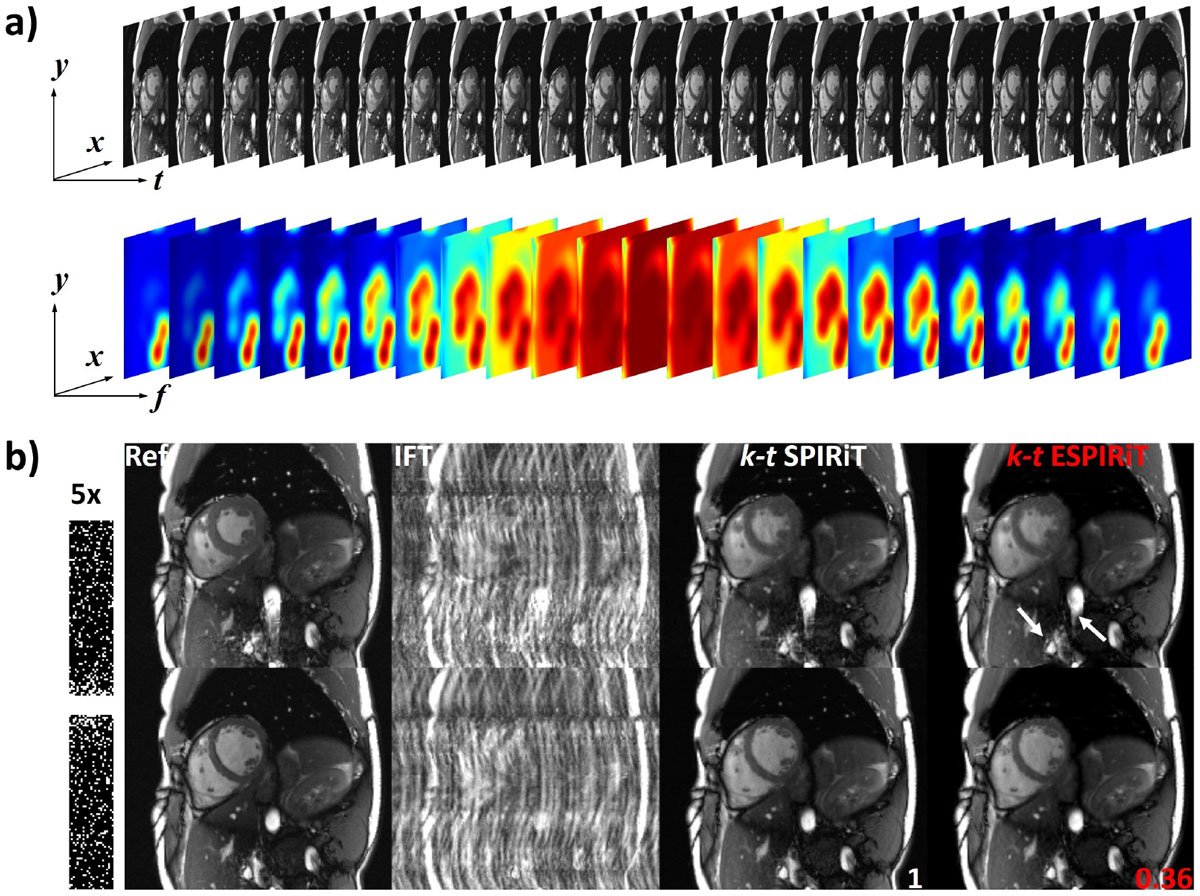
**a) Fully sampled reference** ***x-t*** **image series (top) and** **G** **operator** ***x-f*** **eigenvalue images (bottom) derived from the corresponding 5-fold undersampled data set**. The eigenvalue maps reveal the *x-f* support of the object, and thus, can be incorporated before **S**_*x,f*_matrix (composed of corresponding eigenvectors) multiplication as an additional diagonal weighting matrix multiplication to suppress unwanted temporal frequencies. **b)** Systolic (top) and diastolic (bottom) frames of reference data and IFT, *k-t* SPIRiT and *k-t* ESPIRiT reconstructions from 5-fold undersampled *k-t* data. The sampling pattern in the temporal phase encode plane is depicted on the left. Reconstruction times relative to *k-t* SPIRiT are also shown, i.e. *k-t* ESPIRiT was approximately three-times faster. Arrows mark suppressed image artifacts present in the systolic reference and *k-t* SPIRiT image.

Figure 2a illustrates the fully sampled reference *x-t* image and *x-f* eigenvalue maps derived from **G**. Figure 2b compares direct Fourier transformed (IFT), *k-t* SPIRiT and *k-t* ESPIRiT reconstructed systolic and diastolic frames relative to the reference data.

## Conclusions

Eigendecomposition of the *k-t* SPIRiT operator has been proposed and implemented to reduce computational costs. In-vivo experiments showed equivalence of *k-t* SPIRiT and *k-t* ESPIRiT, and up to 3-fold reconstruction time savings of the proposed relative to the standard method. Thus, further advances towards feasible reconstruction times for iterative solvers for combined PI and compressed sensing have been provided.

